# Secondary Knee Osteoarthritis due to Neurofibromatosis Type 1 Treated with above the Knee Amputation: A Case Report

**DOI:** 10.1155/2013/782106

**Published:** 2013-07-07

**Authors:** Jay Patel, Jeffrey Whiting, Daniel Jones

**Affiliations:** Orthopaedic Department, Saint Louis University, 3635 Vista Boulevard, St. Louis, MO 63104, USA

## Abstract

*Background*. Neurofibromatosis Type 1 (NF-1) has a variety of associated orthopaedic manifestations that have been previously reported. We report a case of severe, grade 4 knee osteoarthritis (OA) with recurrent subluxation and joint laxity due to multiple extra-articular neurofibromas ultimately treated with Above the Knee Amputation (AKA). *Case Description*. A 39-year-old man presented with multiple neurofibromas and lymphedema leading to degenerative changes of the knee. Conservative treatment failed due to the severity of the knee degeneration and patient discomfort. Likewise, arthroplasty was not possible due to poor bone quality and joint instability. Therefore, AKA was selected to relieve symptoms and provide functional improvement. six months after the procedure the patient has increased functional capacity for ambulation and activities of daily living, as well as significant decrease in pain and discomfort. *Clinical Relevance*. Extra-articular neurofibromas causing severe secondary OA in relatively young patients can be functionally improved with AKA and prosthetic device use.

## 1. Introduction

Neurofibromas are defined as benign peripheral nerve sheath neoplasms comprising of a variety of cell types. They are ill defined and commonly involve nerve fascicles and surrounding soft tissues. This involvement of soft tissues can lead to deformity of associated joints, which can cause valgus and varus laxity, as well as joint capsule compromise. These factors can all predispose a susceptible individual to recurrent subluxation [1]. Recurrent subluxation can lead to secondary OA in this patient population. Surgical approach, soft tissue quality, and joint stability were factors that led to the ultimate, consensual decision of proceeding with an AKA in this particular patient. This paper aims to demonstrate the need for recognition of the long-term musculoskeletal effects of NF-1, as well as describing the surgical decisions and methods taken in regards to a particularly young man with debilitating OA and functional impairment.

## 2. Case Report

A 39-year-old African American male patient with NF-1 presented to the Orthopaedics Department of Saint Louis University with the chief complaint of a 30-year history of left knee instability. He was diagnosed with NF-1 after minor trauma resulted in left knee edema at age 9 and subsequent testing revealed NF-1. As he grew, this swelling also increased. He had progressive difficulty walking, only ambulating one block without aid. He could neither run nor squat. His leg was very uncomfortable and painful, and it required his constant readjustment and reengagement due to recurrent subluxations. He had two osteotomies, one per leg between ages 10 and 15. The patient had no history of any types of fractures, but minor trauma caused recurrent subluxations throughout the years. His most recent trauma was two years ago, when minor stumbling on stairs resulted in excessive subluxation of the tibia. He had no other relevant past medical history. Other relevant family history included NF-1 in his father.

**Figure 1:**
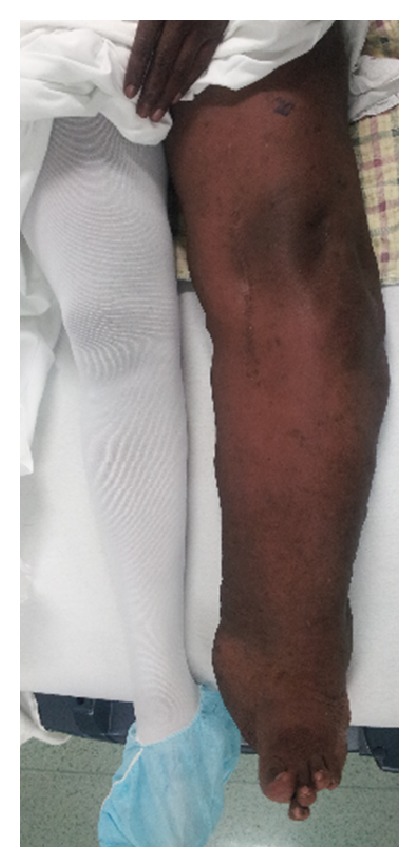
Patient has visible deformity and lymphedema of  left leg.

**Figure 2:**
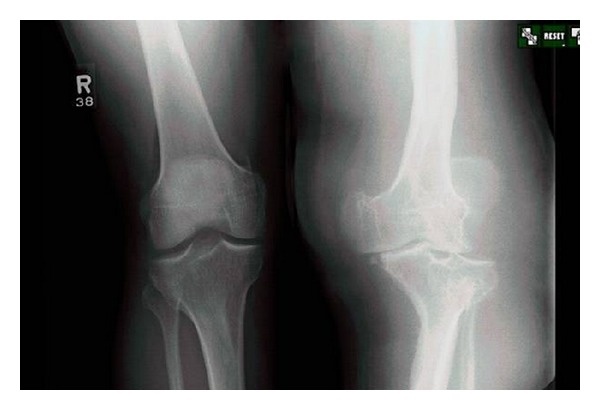
Left knee demonstrates grade 4 OA and varus deformity of  left leg.

**Figure 3:**
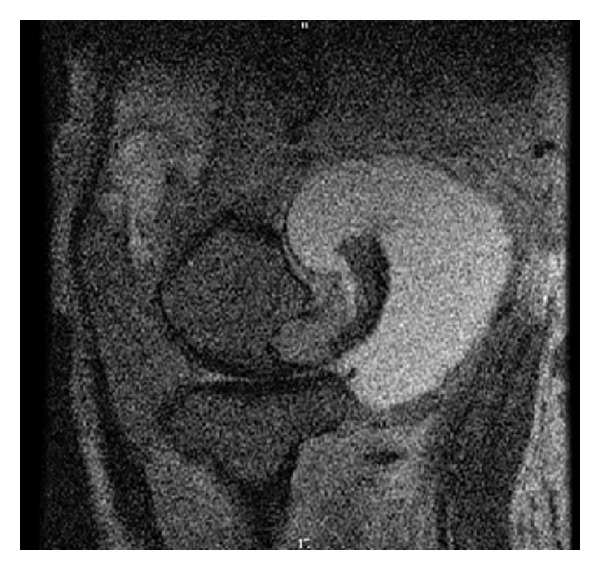
Suprapatellar and posterior knee effusions are visualized with MRI.

**Figure 4:**
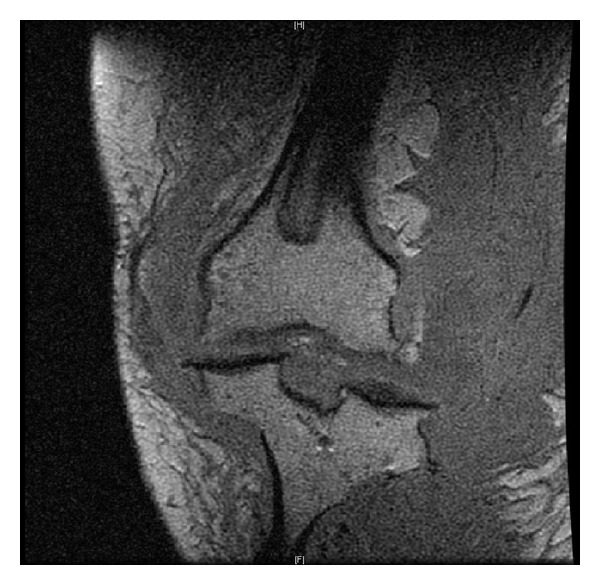
MRI demonstrates bony deformities of distal femur and proximal tibia.

On examination, left leg was significantly larger than the right due to hemihypertrophy and lymphedema. The knee was very unstable, and crepitus with range of motion was present. A large effusion was present posteromedially, and there was minimal ligamentous support (Figure 1). Bilateral knee radiographs revealed a significantly dysplastic and sclerotic left distal femur and left proximal tibia with visible varus deformity. Significant joint space narrowing and medial and lateral compartment bone-on-bone contact were present. Figure 2 provides evidence of a Kellgren-Lawrence grade 4 OA of the left knee. MRI found extensive soft tissue swelling. Two effusions were present: one large suprapatellar effusion and one posterior to the knee measuring 8.2 × 7.3 × 6 cm (Figure 3). Also present were chronic bony deformities of the distal femur and proximal tibia (Figure 4). The posterior cruciate ligament was thinned yet intact. The quadriceps and patellar tendons were intact. The medial collateral ligament was stretched but also intact.

**Figure 5:**
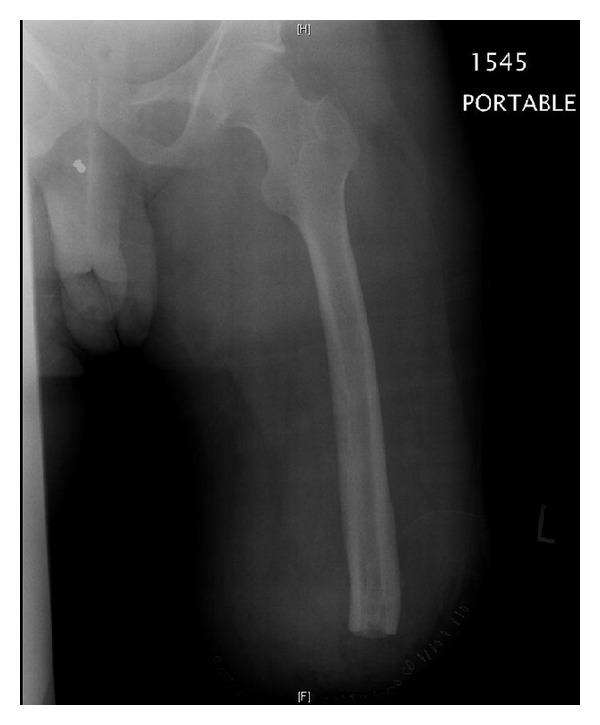
Left femur postamputation.

**Figure 6:**
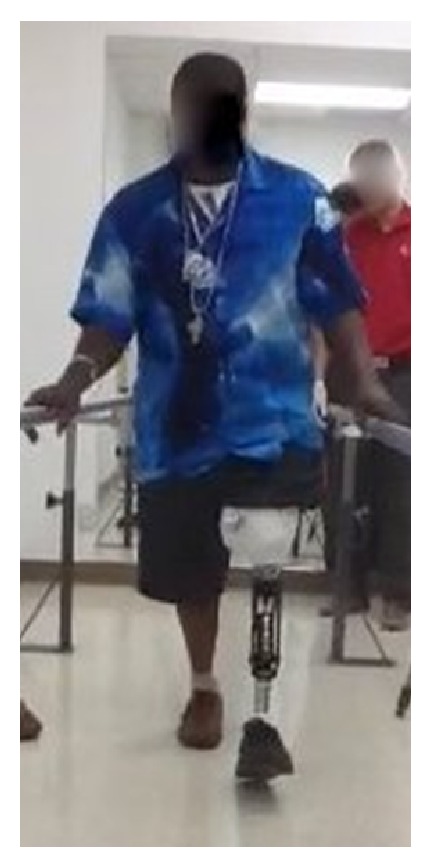
Patient 2 months after amputation, during routine physical therapy.

The operation was performed using a classic transfemoral/supracondylar approach. There were no complications or difficulties during the procedure. The following were notable findings discovered during the operation: most muscles were visibly atrophic, a large neurofibroma was visualized in the tibial nerve prior to dissection, and the patient's lymphedemic leg had resulted in extensive vascular anastomoses and hypervascularization. The left femur postamputation is seen in Figure 5. As of 6 month followup, patient reports minimal discomfort, improved functional capacity and ambulation of more than 2 blocks with no physical aids (other than prosthetic device), and no complications due to surgery other than slight stump bleeding occurring soon after operation, which has not recurred.  Figure 6 depicts patient during initial physical therapy 2 months after amputation.

## 3. Discussion

Neurofibromas have been reported to cause accelerated bone and soft tissue growth [2]. This soft tissue growth and bone involvement can result in a variety of orthopaedic manifestations. Scoliosis is far and away the most common orthopaedic manifestation of NF-1, with 21–49% of patients having some sort of spinal abnormality. Other spinal manifestations that present themselves in NF-1 include abnormalities of the cervical spine and spondylolisthesis. Some proposed etiologies include osteomalacia, intraspinal neurofibromas, and endocrine disturbances. The high prevalence of spinal deformities requires routine screening and increased vigilance for these diagnoses [1, 3, 4]. Another associated orthopaedic manifestation is congenital pseudarthrosis of the tibia (CPT): an anterolateral bowing of the tibia presenting in the first year of life and sometimes even at birth. It is uncommon in the general population, with 1 in 250,000 affected births [5]. CPT is rare: it is found in only 5% of NF-1 patients, but 75% of CPT patients have concurrent NF-1. The anterolateral bowing is associated with spontaneous fracture followed by the development of tibial pseudarthrosis, a common orthopaedic complication in NF-1 [6–8].

The loss of neurofibromin 1 function (the mechanism inherent to NF-1 pathophysiology) can cause generalized metabolic bone disorders. Neurofibromin 1 is also an important regulator in the growth and development of the skeleton (notably joint formation and maintaining bone strength) [9]. Studies have repeatedly verified a general decrease in bone mass in NF-1 patients relative to average bone mass. This is especially apparent in the lumbar spine. One study [10] demonstrated that the level of bone mineral density met the criteria for osteopenia in 48% of patients and osteoporosis in another 25%. Although our patient was not examined for osteoporosis or osteopenia with a DEXA scan, the loss of neurofibromin 1 was undoubtedly involved in his orthopaedic manifestations and presentations.

Abnormal growth patterns are a common feature of  NF-1 patients, being commonly referred to as unilateral segmental hypertrophy (gigantism). Subperiosteal growth and consequent aberrant bone elongation have also been associated with NF-1 [11]. Osteotomies to equalize these aberrant limb lengths achieve both cosmetic and functional improvements for patients [12], and this was performed for our patient during his adolescence.

OA of the knee typically develops bilaterally in the general population. It has been reported as occurring unilaterally in NF-1 in various joints; another case report has demonstrated this unilateral OA of the knee. That particular patient's radiographic presentation was similar in severity to ours (likely grade 3-4 OA); however, that case was in a 55-year-old female with NF-1, and she was only treated with tumor resection due to relative knee stability [13]. OA has more commonly been reported in the hips, which has been associated with recurrent dislocations and treatment with arthroplasty [14–17].

Our patient is unique in the fact that he had no other characteristic orthopaedic manifestations (including spinal deformities, tibial pseudarthroses, or history of fractures) other than deranged bone growth during childhood requiring bilateral osteotomies. He presented with grade 4, unilateral OA with leg deformity, and lymphedema. To our knowledge, no other reports exist of any patients presenting at a similar age with a similar severity of OA as ours. His bone was not conducive to stemmed, constrained knee arthroplasty which would have been required due to the ligament laxity present. Arthrodesis would have been unsuccessful in eradicating his massive painful lymphedema, so an amputation was performed to increase patient quality of life and reduce possible complications of untreated NF-1, including, but not limited to, malignant transformation of neurofibromas and further deterioration of the knee.

## 4. Summary

In the case of this 39-year-old man, his musculoskeletal presentation was abnormal for NF-1: grade 4, unilateral secondary OA is not one of the more commonly reported or studied features of the disease. He had no other history of orthopaedic manifestations other than aberrant limb lengths in his adolescence. This OA (in addition to severe lymphedema due to multiple neurofibromas' presence) was his only functionally limiting symptom. He was fairly young to present with grade 4 OA, but, at that time, little could be done to salvage the knee. Therefore, AKA was proceeded with to alleviate symptoms and provide functional improvement for this patient. As of most recent followup, patient has minimal discomfort, has subjectively increased quality of life, and has increased functional capacity. Had this patient presented at an earlier age, removal of neurofibromas and subsequent arthroplasty may have been possible. Therefore, this case demonstrates the need for vigilance of OA incidence and progression associated with the presence of extra-articular neurofibromas in close proximity to the knee.

## References

[1] Feldman DS, Jordan C, Fonesca L (2010). Orthopaedic manifestations of neurofibromatosis type 1. *Journal of the American Academy of Orthopaedic Surgeons*.

[2] Gutmann DH, Aylsworth A, Carey JC (1997). The diagnostic evaluation and multidisciplinary management of neurofibromatosis 1 and neurofibromatosis 2. *Journal of the American Medical Association*.

[3] Delucia TA, Yohay K, Widmann RF (2011). Orthopaedic aspects of neurofibromatosis: update. *Current Opinion in Pediatrics*.

[4] Zou C, Smith KD, Liu J (2009). Clinical, pathological, and molecular variables predictive of malignant peripheral nerve sheath tumor outcome. *Annals of Surgery*.

[5] Sulaiman AR, Nordin S, Faisham WI, Zulmi W, Halim AS (2006). Residual nonunion following vascularised fibular graft treatment for congenital pseudarthrosis of the tibia: a report of two cases. *Journal of Orthopaedic Surgery*.

[6] Gilbert A, Brockman R (1995). Congenital pseudarthrosis of the tibia: long-term followup of 29 cases treated by microvascular bone transfer. *Clinical Orthopaedics and Related Research*.

[7] Friedman JM, Birch PH (1997). Type 1 neurofibromatosis: a descriptive analysis of the disorder in 1, 728 patients. *American Journal of Medical Genetics*.

[8] Stevenson DA, Birch PH, Friedman JM (1999). Descriptive analysis of tibial pseudarthrosis in patients with neurofibromatosis 1. *American Journal of Medical Genetics*.

[9] Kolanczyk M, Kossler N, Kühnisch J (2007). Multiple roles for neurofibromin in skeletal development and growth. *Human Molecular Genetics*.

[10] Brunetti-Pierri N, Doty SB, Hicks J (2008). Generalized metabolic bone disease in Neurofibromatosis type I. *Molecular Genetics and Metabolism*.

[11] Herrera-Soto JA, Crawford AH, Loveless EA (2005). Ossifying subperiosteal hematoma associated with neurofibromatosis type 1. Diagnostic hesitations: a case report and literature review. *Journal of Pediatric Orthopaedics Part B*.

[12] Szudek J, Birch P, Friedman JM (2000). Growth in North American white children with neurofibromatosis 1 (NF1). *Journal of Medical Genetics*.

[13] Matsukawa Y, Hara H, Ryu J (2009). Unilateral developments of osteoarthritis and Charcot’s joint in a patient with neurofi bromatosis. *Medical Science Monitor*.

[14] Tangsataporn S, Shakib A, Kuzyk PR, Backstein DJ, Gross AE, Safir OA (2012). Secondary hip osteoarthritis due to neurofibroma treated with total hip replacement. *Case Reports in Orthopedics*.

[15] Galbraith JG, Butler JS, Harty JA (2011). Recurrent spontaneous hip dislocation in a patient with neurofibromatosis type 1: a case report. *Journal of Medical Case Reports*.

[16] Odent T, Ranger P, Aarabi M, Hamdy RC, Fassier F (2004). Total hip arthroplasty in a patient with neurofibromatosis type I and recurrent spontaneous hip dislocation. *Canadian Journal of Surgery*.

[17] Haga N, Nakamura S, Taniguchi K, Iwaya T (1994). Pathologic dislocation of the hip in von Recklinghausen’s disease: a report of two cases. *Journal of Pediatric Orthopaedics*.

